# Itraconazole in the Treatment of Aberrantly Active Hedgehog and/or PI3K Recurrent Ovarian Cancer

**DOI:** 10.3390/cancers18091468

**Published:** 2026-05-02

**Authors:** Cynthia S. E. Hendrikse, Noortje Voeten, Phyllis van der Ploeg, Huberdina P. M. Smedts, Hans M. Westgeest, Steven Bosch, Roy I. Lalisang, Birgit E. P. J. Vriens, Anna M. J. Thijs, Sandrina Lambrechts, Ruud L. M. Bekkers, Jurgen M. J. Piek

**Affiliations:** 1 Department of Gynecology and Obstetrics, Catharina Cancer Institute, Catharina Hospital, 5623 EJ Eindhoven, The Netherlands; 2 GROW School for Oncology and Reproduction, Maastricht University, 6211 LK Maastricht, The Netherlands; 3 Department of Obstetrics and Gynecology, Amphia Hospital, 4818 CK Breda, The Netherlands; 4 Department of Internal Medicine, Amphia Hospital, 4818 CK Breda, The Netherlands; 5 Department of Pathology, Eurofins PAMM, 5623 EJ Eindhoven, The Netherlands; 6 Department of Oncology, Maastricht University Hospital, 6229 HX Maastricht, The Netherlands; 7 Department of Oncology, Catharina Cancer Institute, Catharina Hospital, 5623 EJ Eindhoven, The Netherlands; 8 Department of Gynecology and Obstetrics, Maastricht University Hospital, 6229 HX Maastricht, The Netherlands; 9 Department of Obstetrics and Gynecology, Radboudumc, 6525 GA Nijmegen, The Netherlands

**Keywords:** ovarian neoplasms, signal transduction pathways, targeted therapy, hedgehog pathway, phosphoinositide-3-kinase pathway, itraconazole, progression-free survival, precision medicine

## Abstract

Background: Treatment options for recurrent ovarian cancer (OC) are limited, leading to poor prognosis. Targeting tumor-promoting signaling transduction pathways (STPs), such as Hedgehog (HH) and Phosphoinositide-3-kinase (PI3K) STPs, might be an option for treatment. This study evaluates the efficacy of itraconazole as a targeted treatment in HH and/or PI3K active recurrent OC. Methods: We assessed HH and PI3K STP activity in recurrent OC patients. If activity was aberrantly high in either STP, patients received itraconazole treatment, which has been shown to inhibit both HH and PI3K pathways. The primary objective is to compare progression-free survival (PFS) on itraconazole therapy (PFS2) to the PFS on therapy prior to enrolment (PFS1). A PFS2/PFS1 ≥ 1.0 was considered successful. Secondary objectives included side effects, best overall response, one-year survival, and CA125 levels, though this was not a secondary endpoint. Results: Of sixteen patients with successful STP analysis, 93% were eligible for itraconazole therapy. Nine patients started treatment, with a mean duration of 55 days. None achieved a PFS2/PFS1 ratio ≥ 1.0 (mean 0.26, range 0.1–0.7). One patient had radiologically stable disease, while the others experienced disease progression. Side effects were mostly limited to grade 1–2, including fatigue, nausea, dysgeusia, dyspnea, cough, vertigo, and edema. No grade ≥ 3 adverse effects were linked to treatment. One-year survival was 22%. CA125 levels did not correlate with the treatment outcome, but increased rapidly after ceasing treatment. Conclusions: Itraconazole monotherapy for recurrent HH and/or PI3K aberrantly active OC is an ineffective treatment. While CA125 did not correlate with treatment outcome, the rapid increase in CA125 after therapy cessation suggests tumor inhibitory effects.

## Simple Summary

Recurrent ovarian cancer is difficult to treat and new options are urgently needed. This study investigates whether the existing drug itraconazole can slow cancer growth by blocking specific pathways that are involved in the processes of tumor growth and survival. Previous research has suggested that itraconazole has the ability to inhibit these pathways. Patients whose recurrent ovarian cancer tumors showed high pathway activity, measured using an mRNA-based assay, were treated with itraconazole. Disease progression was compared to their previous treatment. Unfortunately, the itraconazole treatment did not show benefit, as almost all patients experienced disease progression and there was no prolonged survival. Although the tumor marker CA125 did not reflect treatment response, its rapid increase after stopping treatment suggests the drug may have had a (temporary) effect on tumor activity. Overall, itraconazole alone does not appear to be an effective treatment, but the findings provide insight for future research into targeted therapies.

## 1. Introduction

Despite aggressive treatment involving surgery and chemotherapy, recurrence of ovarian cancer (OC) is almost always inevitable [1]. Treatment of recurrent OC is generally limited to second- or third-line palliative chemotherapy [2]. Despite the rapid development of targeted therapies for cancer patients, only a few, e.g., poly(ADP-ribose) polymerase (PARP) inhibitors and bevacizumab, have proven to enhance progression-free survival (PFS) in OC patients [3,4].

The poor prognosis may be attributed to the lack of predictive biomarkers to appropriately stratify patients for treatment options [5,6]. Therefore, new stratification approaches are needed to predict the response to targeted therapies. OC heterogeneity, with multiple histological subtypes (i.e., serous, endometrioid, mucinous, clear cell), complicates treatment decisions due to distinct clinical and genomic profiles [7]. Stratification by genomic alterations or protein expression may offer insights into aberrantly active tumor-driving signaling transduction pathways (STPs). However, the presence of these genomic alterations or aberrant protein expression does not always imply aberrantly active STPs, which might be an explanation for the disappointing response rates in clinical trials [5,6,8,9]. The tumor microenvironment is another factor potentially influencing treatment response, but is often disregarded [10]. The tumor microenvironment contains hormones, immune cells and cytokines, which can activate or inhibit STPs, affecting cellular function. Genomic, histopathological and environmental factors influence STPs and ultimately shape the functional phenotype of the cell [11]. Considering that the functional phenotype provides a comprehensive understanding of the cell’s behavior, more insight could potentially improve treatment decisions and outcomes [11,12].

To better reflect functional cell activity, a different strategy was developed using Bayesian network models to determine STP activity from mRNA expression of validated target genes in tumor tissue [12,13,14]. Models are currently available for thirteen pathways involved in oncogenesis [15]. By integrating multiple pathway-specific target genes rather than relying on a single marker, this approach provides a more functional measure of pathway activation. Studies on breast and salivary duct cancers suggest that this mRNA-based assay may aid in selecting patients for pathway-targeted therapies [9,16,17,18].

There are about twelve STPs that are potentially oncogenic when dysregulated [12,19]. The Hedgehog (HH) and Phosphoinositide-3-kinase (PI3K) pathways are major drivers in OC [20,21,22,23]. Dysregulation of these pathways occurs both in the early and late stage of disease [24]. This makes the HH and PI3K pathways two potential therapeutic targets [20,21,22,23].

Itraconazole, registered as an antifungal agent, has shown the ability to inhibit both HH and PI3K pathway activity and may hold promise as a repurposed treatment option [25,26,27,28,29]. Classic HH inhibitors such as vismodegib are direct inhibitors of canonical Smoothened (SMO) on the cell membrane, preventing the activation of the HH signaling cascade. Itraconazole inhibits SMO in an alternative way, impairing downstream GLI activation [30]. Itraconazole shows downregulated ERBB2 mRNA, indicating the inhibition of the PI3K pathway [29]. Its therapeutic potential has demonstrated therapy response in other cancer types as monotherapy, but also in OC in combined chemotherapy treatment with impact on survival [25,26,27,28].

In this study we evaluate the efficacy of itraconazole in OC patients with aberrantly active HH and/or PI3K STP OC as part of a larger prospective study (NCT0345822). Key outcomes include progression-free survival (PFS) on itraconazole treatment compared to PFS on prior therapy (PFS ratio), best overall response, toxicity and survival.

## 2. Materials and Methods

### 2.1. Study Design and Participants

Patients were screened for eligibility in the routine multidisciplinary tumor board (MTB) meeting including seven regional hospitals as part of the Gynecologic Oncologic center south (GOCZ) in the Netherlands or through referral from external hospitals. Patients with recurrent OC, regardless of histology, were eligible if they met one of the following criteria: (1) platinum-resistant OC, (2) refusal of standard therapy, or (3) asymptomatic with elevated CA125 levels (>35 U/mL) after first-line treatment. Additional criteria included radiologically evaluable disease according to RECIST 1.1 criteria, WHO performance status 0-II and the ability to obtain a tumor biopsy with a tumor cell percentage (TCP) ≥ 50% to reduce stromal contamination. Exclusion criteria included concurrent anti-cancer therapy or other malignancies. Eligible patients were referred to the initiating center (Catharina Hospital Eindhoven). The full eligibility criteria, full methods and materials, sample size and plan for analysis are described in the study protocol [31]. This study was reported according to the TREND guidelines (Table S1) [32].

### 2.2. Tumor Sampling and Signal Transduction Pathway Activity Analysis

Obtained biopsy tissue was prepared by a pathologist. Subsequently, RNA extraction, qRT-PCR and measurements of mRNA concentrations were performed. STP scores for seven oncogenic pathways (Mitogen Activated Protein Kinase (MAPK), Androgen Receptor (AR), Estrogen Receptor (ER), HH, Notch, Transforming Growth Factor–β (TGF-β), and PI3K) were determined using OncoSIGNal technology (InnoSIGN Inc., Eindhoven, The Netherlands) to obtain a comprehensive tumor profile and to analyze possible crosstalk mechanisms.

### 2.3. Treatment Decision and Follow-Up

STP activity scores were compared to reference values from healthy fallopian tube epithelium (FTE), the tissue of origin of the majority of OC [24,33]. To correct for natural variation within the samples (*n* = 20), cut-off values for aberrantly high STP activity were determined as two standard deviations above the mean FTE values [24,34]. The MTB evaluated these scores along with clinicopathological data, to determine eligibility for itraconazole therapy. An MTB, compromised of oncologists, gynecologic-oncologists, radiologists, radiation therapists and pathologists, evaluated these scores through a standardized form to help guide treatment selection. The form assessed the score elevation above the cut-off, identified the most likely tumor-driving pathways, and determined the availability of targeted therapies within the study. Alongside the individual’s clinicopathological data, eligibility for itraconazole therapy was determined.

Treatment was initiated with itraconazole 300 mg PO BID, within four weeks of MTB decision. Baseline CT scans were performed within two weeks before treatment initiation. Follow-up consultations occurred at least 1, 3, 6, and 12 weeks after treatment initiation, including laboratory tests and itraconazole toxicity evaluation by CTCAE v5.0. Adverse events were reported from treatment initiation until 12 weeks after ceasing treatment, with the highest grade reported for recurring or worsening events. Radiological assessments followed every 12 weeks.

### 2.4. Dose Reductions and Treatment Cessation

Itraconazole levels were monitored during treatment. Serum levels were evaluated after 1, 3, 6 and subsequently every 12 weeks. A serum level ≥ 6.0 mg/L (sum of serum levels itraconazole and active metabolite hydroxy-itraconazole) was considered potentially toxic in our study population, based on therapeutic drug monitoring studies reporting an increased incidence of adverse effects at itraconazole concentrations of ≥5.0 mg/L [35]. In case of unacceptable toxicity or toxic serum levels, temporary discontinuation and dose reduction took place to 200 mg PO BID. Treatment continued until disease progression, unacceptable toxicity, or death. For research purposes, patients were asked to undergo a secondary biopsy for STP analysis after treatment was ceased to particularly evaluate the HH and PI3K pathway activity and pathway escape mechanisms after treatment.

### 2.5. Outcome Measures

The primary outcome was the PFS2/PFS1 ratio, comparing the PFS of prior treatment (PFS1) to the PFS under itraconazole (PFS2) [36]. PFS2/PFS1 ≥ 1.0 was considered successful. As part of a multi-arm, multistage trial design, in case none of the first eight patients achieve PFS2/PFS1 ≥ 1.0, the treatment arm should be closed due to the lack of efficacy [31]. Secondary outcomes included best overall response by RECIST 1.1, one-year survival, overall survival and adverse effects.

### 2.6. (Statistical) Analysis

Statistical analysis was performed using SPSS version 29 and RStudio version 2023.12.1+402. Fisher-exact and Mann–Whitney U tests were used for categorical and continuous variables respectively. A *p*-value of <0.05 was considered statistically significant.

Using the available data, explorative analysis of CA125 levels was performed. The slope of CA125 change in the period before, during and after treatment was assessed. CA125 levels during subsequent anti-cancer therapy were excluded from analysis.

## 3. Results

### 3.1. Patient Population

**Figure 1:**
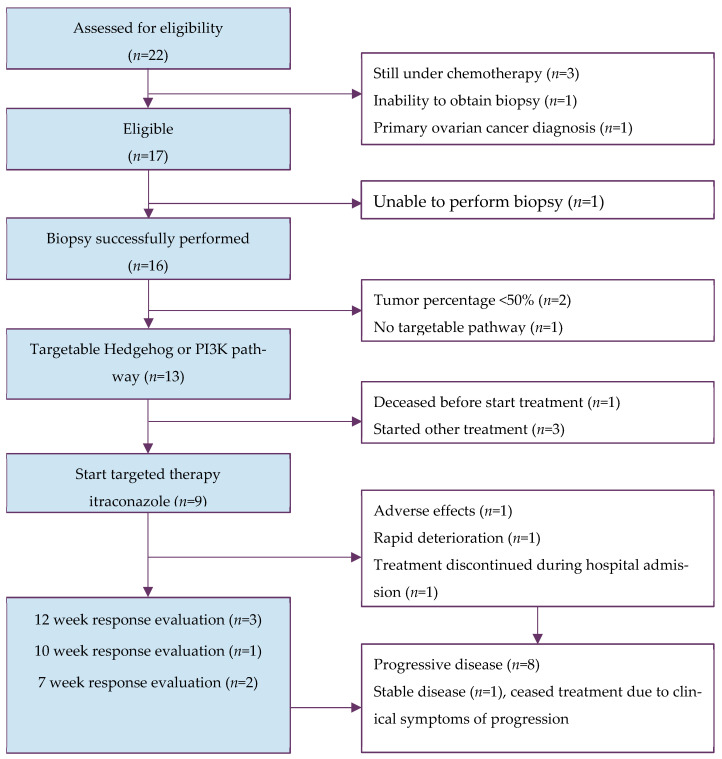
Flowchart of inclusion of patients. Abbreviations: PI3K = Phosphatidylinositol 3-kinase.

A total of 22 patients were assessed for eligibility. Five patients did not meet the eligibility criteria due to active chemotherapy treatment (*n* = 3), inability to obtain a tumor biopsy (*n* = 2) and primary OC diagnosis (*n* = 1) (Figure 1). Biopsies were successful in 16 patients. After pathologist assessment, two patients were excluded due to insufficient TCP. Thus, samples of 14 patients were available for analysis of STP activity. The MTB deemed 13 patients, with an aberrantly active HH and/or PI3K pathway, eligible for targeted therapy with itraconazole. For nine patients itraconazole therapy was initiated; the other eligible patients were excluded due to unexpected death (*n* = 1) and preference to pursue other treatment (*n* = 3). Baseline characteristics are presented in Table 1.

### 3.2. Itraconazole Treatment

Nine patients initiated itraconazole treatment and the average treatment time was 55 days (range 24–85 days). Three patients with recurrent high-grade serous OC ceased treatment prematurely due to unacceptable side effects, rapid clinical deterioration or hospital admission. The median PFS1 was 7.2 months and PFS2 was 1.7 months. The mean PFS2/PFS1 ratio was 0.26 (range 0.1–0.7).

Radiological response was evaluated in seven patients. The best overall response at the first radiological evaluation was stable disease for one patient, and the remaining patients all showed progressive disease (Table 2). For two patients, no radiological evaluation could be performed due to rapid deterioration and early discontinuation of treatment followed by prompt subsequent chemotherapy.

During toxicity monitoring, eight toxic serum levels of itraconazole were observed in six patients. This led to a total of four dose reductions in three patients. One patient experienced unacceptable adverse effects and discontinued therapy after dose reduction, which did not improve symptoms. The remaining patients had toxic serum levels and treatment was ceased due to progressive disease or hospital admission. One patient was incompliant with the itraconazole use after six weeks of treatment. This was confirmed by almost absent itraconazole serum levels six weeks later. Based on the serum levels, there were no other indications for incompliance in other patients.

**Figure 2:**
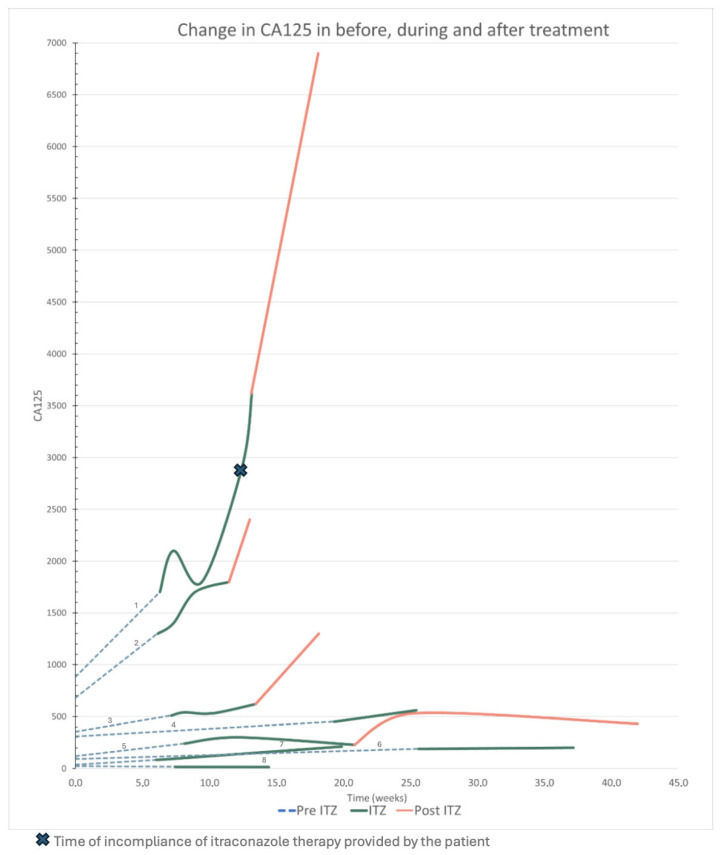
Change in CA125 before during and after treatment with itraconazole. Numbers 1–8 refer to the clinical characteristics described in Table S2.

CA125 tumor marker levels were available from eight patients for analysis. In 50% of the patients, the slope during treatment was more gradual compared to pre-treatment. In one of these patients, CA125 levels decreased (Figure 2) and for three patients the CA125 levels increased faster compared to pre-treatment. Notably, all four patients with available CA125 levels after itraconazole treatment experienced a marked rise in CA125 levels after ceasing treatment with itraconazole.

There was no tumor tissue available for STP analysis after Itraconazole treatment.

### 3.3. Adverse Effects and Serious Adverse Events

Overall, patients reported itraconazole treatment to be tolerable. Common adverse effects were fatigue, nausea, dysgeusia, dyspnea, loss of appetite, cough, vertigo and edema (CTCAE grade 1–2). Grade ≥ 3 adverse effects were related to serious adverse events (SAEs). One patient underwent elective abdominal surgery for a pre-existing enterorectal fistula, complicated by multiple reoperations due to anastomotic leaks and peritoneal infections (CTCAE grade 3). This resulted in death shortly after ceasing therapy (CTCAE grade 5). Another patient was hospitalized for a complicated urinary tract infection, heart failure and atrial fibrillation (CTCAE grade 3). Upon admission, itraconazole serum level was at a toxic level. One patient had rapid progression which resulted in death (CTCAE grade 5). All SAEs were acknowledged by the Medical Research Ethics Committees United and no causal relationship was determined with itraconazole. All adverse effects and SAEs are described in Table 3.

## 4. Discussion

In this study we found HH and/or PI3K pathways are aberrantly active in almost all recurrent OC tumors. Targeted treatment with itraconazole, containing HH and PI3K inhibitory properties, did not result in improved clinical response or increased PFS (PFS2/PFS1 ratio < 1.0). Since this treatment arm in the study did not reach its primary endpoint, further accrual of patients for treatment with itraconazole was discontinued.

Previous studies reported a significantly longer PFS when itraconazole was combined with chemotherapy compared to chemotherapy alone in patients with refractory OC and recurrent clear-cell OC [25,26]. In contrast, our study used itraconazole as monotherapy, achieving stable disease in one patient with a treatment duration of 12 weeks. This may reflect the patient population, which included heavily pretreated individuals with poor prognosis, resulting in a low likelihood for successful treatment response. Additionally, it is important to consider that itraconazole has been reported to inhibit angiogenesis and may resensitize refractory OC by inhibiting the P-glycoprotein efflux pump. These mechanisms could potentially enhance the effectiveness of itraconazole when used in combination with chemotherapy, which may explain the differences in outcomes observed in previous studies compared to our findings with single-agent itraconazole [25,37].

The low efficacy observed could also be due to the involvement of other active tumor-driving pathways. Although all patients had an aberrantly active HH and/or PI3K pathway, in 46% of these patients, there was yet another active pathway (Table S3). The HH pathway is activated through overexpression of the Sonic HH ligand (Shh), loss-of-function mutations of included membrane receptors and gain-of-function mutations of the SMO. The latter is the target for itraconazole and other drugs targeting the HH pathway. Itraconazole binds to SMO on the cell membrane, suppressing its activity. This binding mirrors the situation in absence of ligand Shh where the expression of HH target genes are suppressed [30]. For the PI3K pathway the inhibitory mechanism is more unknown. There is evidence that the PI3K pathway is inhibited through the decreased expression of HER2/ERBB2. Moreover, in multiple studies, a decrease in the phosphorylation of the AKT and S6 protein, both downstream components of the PI3K pathway, was observed [29,38,39]. During treatment with inhibitors targeting the HH and/or PI3K pathways, other oncogenic STPs such as the MAPK pathway may become more active and contribute to tumor progression [40]. Pathway crosstalk and multi-active STPs may be the reason monotherapy with itraconazole is not sufficient to achieve tumor response. Therefore, in line with the treatment strategy of previous studies with positive results, combination therapy targeting multiple pathways might be more effective. Albeit, at the cost of more side-effects.

The optimal dosage of itraconazole as an anti-cancer treatment is not yet determined. Based on the limited previous studies performed with itraconazole as an anti-cancer treatment, the dosage was determined to be 300 mg PO BID [28,41]. To achieve the maximum possible tumor suppressive potential of itraconazole, the dosage may have to be increased. However, this may not be achievable without compromising on the risks of serious adverse effects and toxicity. In our study, six out of nine patients had one or more itraconazole toxic serum levels during treatment. Although most of these patients did not experience serious adverse effects, itraconazole is characterized by dose-dependent toxicity and increasing dosage should only be considered closely monitoring adverse effects and when clinical benefit is reasonably expected [35].

CA125 is a commonly used tumor marker in OC for monitoring treatment response, but is not disease-specific [42]. While increases in CA125 can be attributed to non-cancerous conditions such as abdominal infections, explanations for decreases or stabilization in CA125 levels—aside from treatment response—are not well-documented [43]. There are no established pharmacodynamic interactions between itraconazole and CA125. Although fluctuations in CA125 levels are a known phenomenon, this is unlikely to explain the observed effects. Despite no radiological response in our study population, the more indolent increase or stabilization of CA125 was noted in four (50%) patients. A similar effect was observed in a phase II study where 53% of the patients with biochemically recurrent prostate cancer had a decline in PSA levels [28]. This suggests that itraconazole may have a suppressive effect on tumor activity to some extent, possibly slowing down tumor growth by inhibiting the PI3K and HH STPs.

This is the first prospective cohort study in recurrent OC using functional STP analysis to stratify patients for targeted treatment. Although this technique has shown predictive value in retrospective studies in breast and salivary duct cancer, there are some limitations to consider. Firstly, in the absence of validated cut-off values, we used healthy FTE from post-menopausal women to determine reference values. While this is a reasonable approach, it may not accurately reflect biologically or clinically relevant STP activity. To improve the clinical applicability of this approach, future studies should aim to validate cut-off values in larger cohorts with prospective clinical outcome data. Establishing reference values will be essential to optimize patient stratification.

Secondly, the STP activity profile included seven oncogenic pathways. Although this includes the most important pathways involved in oncogenesis, there may be underlying crosstalk mechanisms from unexamined STPs that are overlooked. Lastly, the tumor after itraconazole treatment could not be evaluated due to unavailability of post-itraconazole biopsies. This is important to gain better understanding in evaluating the success of targeted STP inhibition, STP crosstalk and the development of resistance mechanisms. Cross-talk mechanisms have been reported before between the HH and PI3K, TGF-β and MAPK pathways. The PI3K pathway has interaction with the MAPK, NF-κB and the Wnt pathways [44,45]. Such interactions may enable rapid compensatory activation of alternative survival pathways following inhibition of HH or PI3K signaling, for example through downstream activation of GLI via PI3K or MAPK signaling through upregulation of receptor tyrosine kinase.

To improve the efficacy of targeted therapies in personalized medicine, multiple components play a role, including patient selection, treatment matching, and drug delivery. Future strategies in recurrent OC should therefore focus on improved selection of patients for appropriate therapies. Functional STP tumor profiling, as applied in our study, contributes to more precise identification of patients who are most likely to benefit from pathway-targeted treatments. Furthermore, minimizing adverse effects and considering quality of life in cancer treatment are an important goal from the patient perspective. To support this, the pharmacokinetic properties of therapeutic agents should be considered, and alternative administration or drug delivery approaches may be explored in the future to optimize bioavailability, improve tumor targeting, and improve safety profiles. Nanocarrier-based drug delivery systems represent one such approach. A well-established example in clinical practice is Caelyx, a liposomal formulation of doxorubicin used in recurrent OC treatment [46]. More advanced nanocarrier systems are currently under investigation [47].

Itraconazole alone is insufficiently potent to induce tumor regression in recurrent OC patients. However, this repurposed drug may be promising in other treatment phases. For example, combined with adjuvant chemotherapy during primary treatment, or as maintenance therapy. At these stages, there is less (genomic) heterogeneity induced by subsequent anti-cancer treatments compared to recurrent OC [36]. By inhibiting HH and/or PI3K pathways at an earlier stage, the goal is to prolong disease-free and overall survival. Furthermore, combination therapy with other targeted drugs like MAPK inhibitors might be an option for treatment of women with recurrent OC. Itraconazole could also be combined with other HH inhibitors to potentially gain a stronger inhibitory effect, since targeted HH inhibitory drugs like vismodigib bind to another part of SMO compared to itraconazole [29].

## 5. Conclusions

In conclusion, itraconazole as targeted therapy for aberrantly active HH and/or PI3K recurrent OC did not prolong survival and has very limited clinical benefit.

## Figures and Tables

**Table 1:** Clinicopathological characteristics of included patients treated with itraconazole.

Variable	Patients (*n* = 9)
**Age at diagnosis (mean years, SD)**	63 (6.8)
**Menopausal status (no., %)**	9 (100)
**Histological subtype (no., %)**	
HGSC	7 (78)
LGSC	2 (22)
**FIGO stage at diagnosis**	
IC	1 (11)
IIIC	2 (22)
IV	6 (67)
**Debulking Type**	
Primary	2 (22)
Interval	5 (55)
Other	2 (22)
**Debulking Outcome**	
Complete	8 (89)
Optimal	0 (0)
Incomplete	1 (11)
**Number of recurrences**	
1	5 (56)
2	4 (44)
**Recurrence treatment**	
Chemotherapy + PARPi	2 (22)
PARPi	1 (11)
AHT, chemotherapy	1 (11)
Debulking surgery, chemotherapy	1 (11)
Chemotherapy	1 (11)
Chemotherapy, PARPi, radiotherapy	1 (11)
Chemotherapy, radiotherapy	1 (11)
No treatment	1 (11)
**Progression-free survival 1 (median months)**	7.3
**One-year survival (no patients, %)**	
Survived	1 (11)
Deceased	7 (78)
Censored	1 (11)
**Overall survival (median months)**	7.13

Abbreviations: HGSC = high-grade serous ovarian carcinoma, LGSC = low-grade serous ovarian carcinoma, FIGO = International Federation of Gynecology and Obstetrics, PARPi = PARP inhibitor, AHT = anti hormonal therapy.

**Table 2:** Best overall response by RECIST 1.1.

No. of Patients (*n* = 7)	Weeks of Treatment at First Radiological Response Evaluation	Best Overall Response During PFS2
1	4	PD
2	7	PD
1	10	SD
3	12	PD

Abbreviations: PD = progressive disease, SD = stable disease.

**Table 3:** Adverse events graded by CTCAE v5.0 during itraconazole treatment and up to 12 weeks after ceasing treatment. The reported numbers represent the number of affected patients, with adverse events classified by the highest grade experienced during the study period.

Adverse Event	Grade 1	Grade 2	Grade 3	Grade 4	Grade 5	Total Patients Affected
Fatigue	3	3	-	-	-	6
Nausea	5	-	-	-	-	5
Anorexia	3	2	-	-	-	5
Dysgeusia	3	1	-	-	-	4
Dyspnea	2	2	-	-	-	4
Cough	3	-	-	-	-	3
Vertigo	3	-	-	-	-	3
Edema limbs	2	-	1	-	-	3
Headache	2	-	-	-	-	2
Diarrhea	1	-	1	-	-	2
Flu-like symptoms	1	-	1	-	-	2
Pain in extremity	2	-	-	-	-	2
Atrial fibrillation	-	1	1	-	-	2
Weight change	1	-	-	-	-	1
Vomiting	1	-	-	-	-	1
Blinking	1	-	-	-	-	1
Abdominal pain	-	1	-	-	-	1
Hot flashes	1	-	-	-	-	1
Hyperhidrosis	1	-	-	-	-	1
Gum infection	1	-	-	-	-	1
Dyspepsia		1	-	-	-	1
Gastrointestinal anastomotic leak	-	-	1	-	-	1
Peritoneal infection	-	-	1	-	-	1
Death NOS	-	-	-	-	1	1
Disease progression	-	-	-	-	1	1
Urinary tract infection	-	-	1	-	-	1
Non-cardiac chest pain	1	-	-	-	-	1
Heart failure	-	-	1	-	-	1
Urticaria	1	-	-	-	-	1
Dehydration	1	-	-	-	-	1
Polyneuropathy	1	-	-	-	-	1
Vaginal discharge	1	-	-	-	-	1
Sinus bradycardia	-	1	-	-	-	1
Hypokalemia	-	1	-	-	-	1
Urinary incontinence	1	-	-	-	-	1
Dry eye	1	-	-	-	-	1
Flatulence	1	-	-	-	-	1
Bloating	1	-	-	-	-	1
Diplopia	1	-	-	-	-	1
Urinary frequency	1	-	-	-	-	1
Papular rash	1	-	-	-	-	1
Pleural effusion	-	1	-	-	-	1
Ileal obstruction	-	1	-	-	-	1

All grade 3–5 events were reviewed by the Medical Ethical board and no causal relationship with the treatment was established. Abbreviations: NOS: not otherwise specified.

## Data Availability

The data generated during analysis in the current study will be available upon request.

## Supplementary Materials

The following supporting information can be downloaded at: https://www.mdpi.com/article/10.3390/cancers18091468/s1, Table S1: TREND-22 checklist; Table S2: Supporting clinical information for Figure 2; Table S3: Aberrant Signal Transduction Pathways.

## Abbreviations

The following abbreviations are used in this manuscript:

OC	Ovarian Cancer
STPs	Signal Transduction Pathways
HH	Hedgehog
PI3K	Phosphoinositide-3-kinase
PFS	Progression-free survival
PFS1	Progression-free survival on therapy prior to enrolment
PFS2	Progression-free survival on itraconazole therapy
PARP	Poly(ADP-ribose) Polymerase
mRNA	Messenger RNA
SMO	Smoothened
MTB	Multidisciplinary Tumor Board
GOCZ	Gynecologic Oncologic center south
RECIST	Response Evaluation Criteria in Solid Tumors
WHO	World Health Organization
TCP	Tumor cell percentage
MEC-U	Medical Research Ethics Committees United
CCMO	Central Committee on Human Research
qRT-PCR	Quantitative Reverse Transcription Polymerase Chain Reaction
MAPK	Mitogen Activated Protein Kinase
AR	Androgen Receptor
ER	Estrogen Receptor
TGF-β	Transforming Growth Factor–β
FTE	Fallopian Tube Epithelium
PO BID	Per os twice daily
CTCAE	Common Terminology Criteria for Adverse Events
SAE	Serious Adverse Event
Shh	Sonic Hedgehog
SD	Stable disease
PD	Progressive disease
HGSC	High-Grade Serous Carcinoma
LGSC	Low-Grade serous Carcinoma
AHT	Anit-hormonal Therapy
FIGO	Internation Federation of Gynecology and Obstetrics
NOS	Not Otherwise Specified
