# Supplementary material for: Itraconazole in the Treatment of Aberrantly Active Hedgehog and/or PI3K Recurrent Ovarian Cancer
Source: Cancers (Basel). 2026 May 2;18(9):1468. doi: 10.3390/cancers18091468 (PMC13163028; doi:10.3390/cancers18091468)
Supplement: Supplementary file 1 [file cancers-18-01468-s001.zip › Table S2. supporting clinical information fig. 2.pdf]

Table S2. Table with supporting individual clinical information in relation to the CA-125 changes described in Figure 2.

Patient number in fig 2.	Tumor histology	Treatment prior to Itraconazole treatment
1	HGSC	Chemotherapy
2	HGSC	Chemotherapy and PARPi
3	HGSC	PARPi
4	LGSC	Chemotherapy
5	HGSC	Chemotherapy
6	LGSC	Chemotherapy and radiotherapy
7	HGSC	Chemotherapy
8	HGSC	Radiotherapy

HGSC= High-Grade Serous Ovarian Cancer, LGSC= Low-Grade Serous Ovarian Cancer, PARPi= Poly ADP-ribose polymerase inhibitor
