# Supplementary material for: Itraconazole in the Treatment of Aberrantly Active Hedgehog and/or PI3K Recurrent Ovarian Cancer
Source: Cancers (Basel). 2026 May 2;18(9):1468. doi: 10.3390/cancers18091468 (PMC13163028; doi:10.3390/cancers18091468)
Supplement: Supplementary file 1 [file cancers-18-01468-s001.zip › Table S3. Aberrant Signal Transduction Pathways.pdf]

Table S3. Aberrant Signal Transduction Pathways (STP) for each patient determined as a value of at least two standard deviations above the mean healthy fallopian tube epithelium values.

**Aberrant STP**	**No. of patients (%)**
HH	4 (28,5)
MAPK, TGF-β, HH	3 (21,4)
HH, PI3K	2 (14,3)
HH, TGF-β, PI3K	1 (7,1)
HH, TGF-β	1 (7,1)
HH, MAPK	1 (7,1)
PI3K	1 (7,1)
TGF-β	1 (7,1)

Abbreviations: MAPK: Mitogen Activated Protein Kinase, AR: Androgen receptor, ER estrogen receptor, HH: Hedgehog, TGF- β: transforming growth factor- Beta, PI3K: Phosphoinositide-3-Kinase
